# Biographical continuation: recovery of stroke survivors and their family caregivers in Taiwan

**DOI:** 10.1017/S1463423623000610

**Published:** 2024-01-05

**Authors:** Zih-Yong Liao, Elaine Haycock-Stuart, Susanne Kean

**Affiliations:** 1 National Center for Geriatrics and Welfare Research, National Health Research Institutes, Yunlin 63247, Taiwan; 2 Nursing Studies, School of Health in Social Science, The University of Edinburgh, Edinburgh EH8 9AG, UK

**Keywords:** dyads, ethnography, life transitions, long-term care, rehabilitation, status passage

## Abstract

**Aim:**

To explore the experiences pertaining to long-term care services from the perspectives of dyads of stroke survivors and their family caregivers in indigenous and non-indigenous communities.

**Background:**

Stroke occurrence is a life-changing event associated with quality of life for stroke survivors and their families, especially those who provide primary support. Indigenous people are more likely to experience a stroke at a younger age and have a higher likelihood of hospitalisation and death due to health disparities. Few studies have investigated family dyads or indigenous populations to understand their experiences of coping with changed body-self and to contextualise their reintegration into communities post-stroke.

**Methods:**

Ethnographic fieldwork over nine months in 2018–2019 with indigenous, urban-based, and non-indigenous populations, resulting in 48 observations and 24 interviews with 12 dyads in three geo-administrative communities.

**Findings:**

The post-stroke recovery trajectory is illuminated, delineating the dyads’ life transitions from biographical disruption to biographical continuation. The trajectory is shaped by seven states involving four mindsets and three status passages. The four mindsets are sense of loss and worry, sense of interdependence, sense of independence, and wellbeing state. The status passages identified in this study are acceptance, alteration, and identification. A community-based and family-centred long-term care system, aligning with medical healthcare and community resources, underpinned each dyad’s biographical continuation by: (1) providing rehabilitation that afforded time and space for recovery adaptation; (2) acknowledging the individuality of family caregivers and helping to alleviate their multitasking; and (3) reintegrating stroke survivors into their communities. Key to determining the quality of recovery for the indigenous participants was their reintegration into their native community and regaining of identity. Therefore, integrating post-stroke care into various care contexts and incorporating indigenous-specific needs into policymaking can support dyads in adapting to their communities.

## Introduction

Stroke is a sudden, disruptive incident that often leaves stroke survivors and their family caregivers struggling with physical changes and recovering from a changed body and new emerging perception of the self (Young et al., 2014). According to the World Health Organization (2019), stroke ranks third in the global leading causes of disability-adjusted life years (DALYs), representing a time-based measure that combines years of life lost due to premature mortality and time spent living with a disability. In Taiwan, the DALYs loss from stroke increased from 992.1 in 2010 to 1360.8 (age-sex standardised DALYs loss/100,000 people) in 2017 (Venketasubramanian et al., 2017; Wu et al., 2021). Furthermore, indigenous people in Taiwan have a higher prevalence of stroke compared to their non-indigenous counterparts (Lin et al., 2021). The long-term care (LTC) system in Taiwan, established to address the needs of individuals and their families living with long-term health conditions, involves various services such as home-based rehabilitation, transportation, and residential care services (Ministry of Health and Welfare, 2016). The LTC system offers services for the following people: (1) individuals over the age of 65; (2) individuals of all ages with LTC needs, as defined by activities of daily living and instrumental activities of daily living; (3) mountain-region and plain-region indigenous people over the age of 55; (4) individuals aged over 65 with frailty; and (5) individuals with dementia aged over 50 (Ministry of Health and Welfare, 2016). Due to the health disparity evidenced by the nearly eight-year shorter life expectancy of indigenous groups in Taiwan (Ministry of the Interior, 2019), these groups can access LTC services ten years earlier (at age 55) than the general population (at age 65). This study extends the understanding of the LTC system’s support of dyads (individuals surviving a stroke event and their family caregivers) from different ethnic and cultural backgrounds.

## Background

### Dyadic experiences of post-stroke life

It is common for stroke survivors to require support to manage residual stroke complications and subsequent life transitions. A narrative inquiry study by Nasr et al. (2016) recruited five stroke survivors and three family caregivers in the United Kingdom (UK). Their narratives described how bodily changes influenced their perception of self and resulted in a stressful situation relating to changes in the familial relationship. ‘Family’ was illustrated as a lifeline, highlighting its significance as the main care support system for stroke survivors (Kitzmüller et al., 2012). In a study by Ostwald et al. (2009), reliance on family support was found to affect stroke survivors’ and family caregivers’ quality of life. The study applied the Perceived Stress Scale to investigate the psychological state of 159 American stroke survivors and caregivers at discharge and at three, six, nine, and twelve months post-discharge. While the stroke survivors’ stress and depression levels decreased over time, this was not observed in family caregivers. This suggests that while stroke survivors may benefit from healthcare interventions, such interventions may not be sufficient to address the needs of their caregivers.

### Insufficient dyadic intervention for family caregivers

Reciprocity in familial relationship and reliance on care support connect the stroke survivor and family caregiver as a unit, a dyad, when providing healthcare and education for post-stroke care (Kitzmüller et al., 2012). Bakas et al. (2014) critically analysed five caregiver intervention studies and five caregiver-stroke survivor dyad intervention studies. The dyadic intervention studies targeted the stroke survivor and family caregiver as a unit of active participants when applying interventions (Bakas et al., 2014). However, dyadic interventions tend to focus on the functionality of the family caregiver, placing emphasis on the preparation of caregiving skills while providing insufficient psychosocial preparation. The lack of balance in terms of attention to family caregivers’ individual needs is also reflected in the study of Lamontagne et al. (2019), who adopted a phenomenological approach using focus groups to understand the continuity of care post-stroke for 37 stroke survivors and 31 family caregivers in Quebec, Canada. The results suggest that the family caregivers prioritised the stroke survivors’ needs and compromised their own personal needs. The dyadic intervention, which mainly focuses on stroke survivors’ care needs for physical recovery, has neglected the fact that the changed body also profoundly impacts the family caregiver’s life (Bakas et al., 2014; Nasr et al., 2016).

### Disparity between indigenous and non-indigenous populations

Studies indicate that indigenous people are generally more likely to experience stroke at a younger age and have a higher likelihood of hospitalisation and death (Gardiner et al., 2021; Santos et al., 2022). These disparities may be attributed to differences in lifestyle, socioeconomic status, and access to healthcare. For instance, a cross-sectional study by Lin et al. (2021) found that indigenous Taiwanese have higher rates of metabolic syndrome, obesity, and unhealthy lifestyle behaviours than their majority counterparts. The prevalence of metabolic syndrome is a significant predisposing factor to stroke and cardiovascular disease (Guembe et al., 2020). Furthermore, ethnic minorities have poorer access to key stroke investigations and interventions, such as prescribed anticoagulants, and experience worse outcomes (Thompson et al., 2022). Narratives from indigenous stroke patients and healthcare providers suggest that co-morbidities, conflicting priorities, inadequate or inflexible services, and transport issues exacerbate the challenges of changing life roles post-stroke (Kelly et al., 2022). Effective communication and involvement of family members need to be addressed to establish a shared understanding of post-stroke care (Kelly et al., 2022).

Few studies have recruited participants from dyads to gain insights into dyadic experiences of coping with residual complications and life transitions post-stroke. Furthermore, limited information is available regarding the views of dyads from the indigenous population. Therefore, it is significant to explore the post-stroke experiences and healthcare needs of stroke survivors and family caregivers from different contexts.

### The study

#### Aims

This study aims to understand the experiences of post-stroke life and engagement with LTC services based on the dyadic experiences of stroke survivors and family caregivers from the perspectives of indigenous and non-indigenous people in Taiwan, reflecting participants with different sociodemographic backgrounds. The focus of this article is on a post-stroke recovery trajectory and specifically addresses the following question:

- What is the post-stroke recovery trajectory of indigenous and non-indigenous people when engaging with the current LTC system?

#### Design

The study design follows a focused ethnographic approach, exploring dyads of stroke survivors and family caregivers’ experiences of engaging with LTC services in communities and analysing non-indigenous, mountain-based, and urban-based indigenous populations’ experiences of receiving long-term care services post-stroke (Liao, 2021). This doctoral study was conducted by the first author, LZY, with guidance from her PhD supervisors, the co-authors. The research team acknowledged potential preconceptions that could influence the study’s rigour due to LZY being a nurse from the mainstream population. LZY recorded her interactions with the dyads and care providers in the fieldnotes, writing in the first person. LZY kept a reflexive diary to self-reflect her role as well as capture the transition and tension of viewpoints, strengthening the analysis process. This focused ethnographic approach allowed me (LZY) to reside with the research participants, ascertain their experiences of LTC services, and make sense of their meanings when among a group of people in a socially constructed situation (O’Reilly, 2012; Madden, 2017). Non-participant observation allowed me to uncover interactions between care providers and care recipients and understand contextualised data in their natural state, such as the home setting (Swanwick, 1994). Semi-structured interviews enabled me to ask more specific questions, thus extending insights from informal or opportunistic conversations during the observation. The combination of these methods provided rich and nuanced data that enabled me to delve into the life patterns and healthcare needs of individuals post-stroke. Data collection lasted nine months, from August 2018 to April 2019. This focused ethnography was presented according to the consolidated criteria for reporting qualitative research (COREQ) (Tong et al., 2007) (Supplementary file 1).

#### Participants and Recruitment

This study recruited a total of 12 dyads of stroke survivors and family caregivers, consisting of mountain-based, urban-based or non-indigenous participants from three different geographical areas to understand their post-stroke experiences. Each group had four dyads. The inclusion criteria for the dyads were: (1) stroke survivor and main carer; (2) aged 20 years or older; (3) receiving LTC service in community-dwelling settings; (4) able to communicate verbally; and (5) no cognitive impairment. Exclusion criteria were if either member of a dyad (1) declined to participate or (2) had experienced mental disorders, as recalling the stroke experience may induce discomfort and exacerbate their mental stability. Potential participants’ capacity to consent was determined during the recruitment process by their capability to answer questions, including the study purpose, right to ask for a suspension or withdrawal, and decision to keep or remove data following withdrawal. Only those who completed the questions on their own were recruited. The inclusion criteria for the mountain-based, urban-based, and non-indigenous populations were based on their identity and residence, as outlined in Table 1.

**Table 1.** Study participants and fieldwork communities

Participant group	Mountain-based indigenous population	Urban-based indigenous population		Non-indigenous population
Dyad of stroke survivor and family caregiver (n)	4	4		4
Inclusion criteria for dyads	Stroke survivor and main carer; Aged > 20 years^*a*^; Receiving LTC service in community-dwelling settings; Able to communicate verbally; No cognitive impairment			
Exclusion criteria for dyads	Either member of a dyad declined to participate or had experienced mental disorders			
Inclusion criteria for *Identity:* qualify as Indigenous Peoples^*b*^ *Residence:* live in an indigenous administrative region^*c*^	Indigenous identity (+)^*d*^ Indigenous residence (+)	Indigenous identity (+) Indigenous residence (−)		Indigenous identity (−) Indigenous residence (−)
Fieldwork community (n)^*e*^	Mountain-based indigenous administrative township (four dyads)	Post-disaster community^*f*^ (two dyads)	Non-indigenous administrative region in urban plains (two dyads)	Non-indigenous administrative region in urban plains (four dyads)

a 20 was the legal age of majority before 2023 in Taiwan; it became 18 in 2023.

b Qualification of indigenous peoples is based on the Status Act For Indigenous Peoples (Council of Indigenous Peoples, 2001).

c Recognition of indigenous administrative regions is based on the Council of Indigenous Peoples (2002).

d (+) fulfils the criterion, (−) does not fulfil the criterion.

e Fieldwork communities are distinguished by geo-administrative regions, which refer to the geographical and administrative characteristics applied to describe the fieldwork sites.

f The post-disaster community where this study recruited urban-based indigenous participants is located in the region adjacent to the mountain-based indigenous administrative township. It was established as a safer location for households affected by the typhoon destruction to resettle in 2009.

I recruited participants from three different types of communities in three different geo-administrative regions, using combined sampling strategies. I began with purposive sampling to select participants who could share their post-stroke experiences expressively and reflexively, enabling a more expansive view and more nuanced understanding of LTC-receiving experiences (Spradley, 1979; Bernard, 2011). As indigenous people are a hard-to-reach population, I adopted snowball sampling based on the social acquaintances I had established in communities, which linked me to further potential participants. The four urban-based indigenous dyads were recruited from two types of community: one was situated in a non-indigenous administrative region in the urban plains (the same type of community where I recruited the non-indigenous group); the other was a post-disaster community in a township adjacent to a mountain-based indigenous administrative township (Table 1). The mean age of the stroke survivors in the mountain-based indigenous group was 79 years, 66.35 years in the non-indigenous group, and 56.25 years in the urban-based indigenous group. Among the 12 dyads, six comprised spousal caregivers, five involved adult children, and one dyad involved a sibling assuming the caregiving role (Table 2).

**Table 2.** Biographical details of the participants

Code	Pseudonym	Role	Age	Relationship
Mountain-based indigenous population				
A1	Andrew	S	73	Husband
	Georgia	F	72	Wife
A2	Bridget	S	84	Mother
	Deborah	F	61	Daughter
A3	Yuri	S	83	Mother
	Molly	F	40	Daughter-in-law
A4	Novia	S	76	Mother
	Beryl	F	38	Daughter-in-law
Urban-based indigenous population				
U1	Linda	S	52	Younger sister
	Prima	F	57	Elder sister
U2	Ella	S	62	Wife
	Bob	F	63	Husband
U3	Hank	S	64	Father
	Ivy	F	30	Daughter
U4	Dora	S	47	Cohabitant (Female)
	Maxwell	F	58	Cohabitant (Male)
Non-indigenous population				
N1	Julian	S	43	Husband
	Ginger	F	36	Wife
N2	Hobart	S	68	Husband
	Clara	F	64	Wife
N3	Leonard	S	75	Father
	Louis	F	50	Son
N4	Driscoll	S	79	Husband
	Delia	F	71	Wife

Abbreviation: S = stroke survivor, F = family caregiver.

#### Ethical considerations

I assert that all procedures contributing to this work comply with the ethical standards of the relevant national and institutional guidelines on human experimentation, the Research Ethics Committee (REC) at the University of Edinburgh (July 2018) in the UK, the REC at National Cheng Kung University (June 2018), the Council of Indigenous People (COIP) (July 2018) in Taiwan, and with the Helsinki Declaration of 1975, as revised in 2008. Additional ethical approval from the COIP was required to involve vulnerable indigenous populations. The participants received study details four days before seeking consent, giving them time to consider their involvement in interviews and observations. The data collection exploring post-stroke perceptions of receiving LTC was performed by a female registered nurse (LZY). For the observation, written consent was obtained from the LTC providers, and verbal consent was sought from the dyads. Consent was obtained from potential dyads before initial contact and re-confirmed before each observation. If other household members enquired about the study, I explained my role and purpose in data collection. After providing this information, I left the decision of whether to participate in the study up to the household members. Written consent was obtained prior to individual interviews from each stroke survivor and their family caregiver. Data were anonymised to ensure confidentiality. I did not disclose any perceptions or complaints from the LTC recipients to ensure the quality of their services was not negatively impacted.

#### Data collection

Non-participant observations (48 sessions) and semi-structured individual interviews (24 sessions) were used to collect empirical data from 12 dyads. In each population group, I observed the care received by six to eight households: four observations for each dyad. As I was shadowing the LTC providers, the exact duration of non-participant observation depended on the LTC session, which generally ranged from 1 to 1.5 h. Fieldnotes were written on-site in simple sentences and refined to note further details only after the observation. Initially, these reflected a general perspective, such as housing environments and interactions between care providers and recipients. Over time, they became more complex and focused, with more selective observations of particular services. This observation period allowed me to identify four dyads for interviews in each geographical setting. Interviews took approximately 45 min and were based on a semi-structured interview topic guide (Supplementary file 2). Data collection stopped when a sufficient depth of data was obtained to describe fully the emerging phenomenon (Fossey et al., 2002; Vasileiou et al., 2018).

#### Data analysis

The data set included 48 observational fieldnotes and 24 individual interview transcripts; they were uploaded into NVivo 12 Pro (Lumivero, 2017). Participants’ excerpts are quoted in the order of pseudonym, identity, role, and relationship between the dyad. Data analysis was initially inductive and involved the following analytical processes: (1) initial and descriptive coding of empirical data; (2) code mapping for phenomenon emergence; and (3) data interpretation through application of abductive reasoning. I focused on the category of ‘New pattern of life’ from the observational notes and ‘Post-stroke recovery trajectory’ from the interview transcripts to ascertain the post-stroke recovery trajectory.

**Figure 1.**
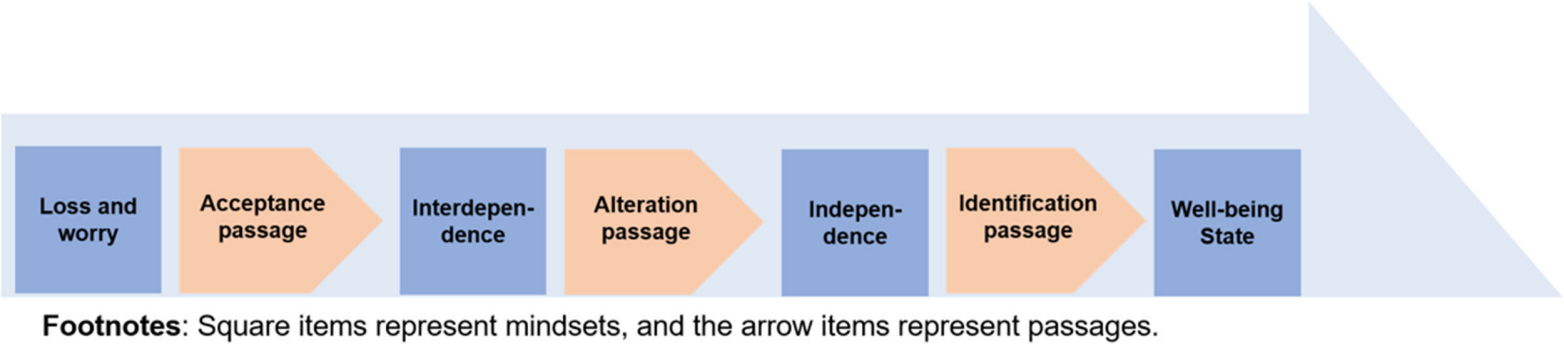
Post-stroke recovery trajectory.

To understand the correlation of the codes and contextualise new patterns of life following stroke occurrence, codes were integrated into the text, mapping onto a post-stroke recovery trajectory (Table 3). This trajectory emerged (Figure 1) as I clustered the research participants’ self-perceived life status and arranged the sub-categories in sequence to bring meaning to the data (Anfara, 2008). The figure shows the stories of the 12 dyads charted in the form of a post-stroke recovery trajectory, which helped contextualise dyadic embodiments of life transitions. To theorise the data, I employed the concepts of biographical disruption (Bury, 1982) and status passage theory (Glaser and Strauss, 2011). Biographical disruption explores the interplay between biomedical illness trajectory and psychosocial trajectory. It encompasses the changes experienced in the body-self due to chronic illness and long-term conditions, impacting one’s lived entity and social identity (Bury, 1982). Status passage theory conceptualises dynamic life transitions and biographical flow by exploring the presence or absence of properties in the social context (Glaser and Strauss, 2011).

**Table 3.** Results in the category of post-stroke recovery trajectory

Category	Sub-category	Codes
Post-stroke recovery trajectory	Sense of loss & worry	Physical incapability
		Distressed about role transformation
	Acceptance passage	Hope to return to normal life
		Rehabilitation is important
	Sense of interdependence	Time was combined with family caregiver
		Life was combined with family caregiver
	Alteration passage	Expect the stroke survivors can be more independent
		Expectation for a new relationship
		Engage with the LTC system
	Sense of independence	Being independent of overcoming challenges
		Me time to do my matters
	Identification passage	Applying self-efficacy
		Establishing self-identity
	Wellbeing state	Think positively
		Feeling good

#### Rigour

To ensure the research’s trustworthiness, I applied six strategies adapted from Lincoln and Guba (1985) and Morse (2015): prolonged engagement, stepwise coding system, triangulation, peer debriefing, reflexive awareness of researcher bias, and thick description. These strategies ensured the rigour of the study. My prolonged engagement in the field built rapport, and writing in first person during field note-taking acknowledged my participatory role in this ethnography. My reflexive diary and the neutral viewpoint from my PhD supervisors as peer debriefers also served as reminders of my active role in this research, which enabled me to address the confirmability.

## Results

The post-stroke recovery trajectory was shaped by seven states, containing four mindsets and three status passages (Figure 1). The four mindsets were sense of loss and worry, sense of interdependence, sense of independence, and wellbeing state. This trajectory proceeded from one mindset to the next under the status passages of acceptance, alteration, and identification. Below, I elaborate on different life transitions along this trajectory using participants’ interview excerpts.

### Sense of loss and worry (mindset 1)

A sense of loss was reported by all stroke survivors in terms of their loss of control over their familiar bodies and fundamental transformation in social role. Andrew, for example, felt distressed when his existing role as a breadwinner in the household was disrupted:

*‘Sometimes, sometimes I felt unwell in my mind… (in terms of) the daily living condition of myself. Certainly, there are barriers in my life. I couldn’t work and couldn’t make money.’ (Andrew, indigenous stroke survivor)*

Worrying about physical incapability and unpredictable accidents resulted in family caregivers adjusting their life development and daily routine to cope with the care needs of stroke survivors. One family caregiver, Delia (wife), described how her daughter adjusted her life to care for her father after his stroke:

*‘My daughter asked for leave from her husband and mother-in-law and came back to care for him (the stroke survivor) for six months. She left her children to her husband and mother-in-law. […] She was an illustrator. But eventually, she had to quit because she was too busy to keep up with the deadlines.’ (Delia, non-indigenous family caregiver, wife)*

Family support was the preferred and foremost resource when people were undergoing life crises. This family caregiving responsibility stemmed from the belief in filial piety and reciprocity in the Taiwanese community, as Louis expressed:

*‘People are all gaining in age. I also need my son to look after me when I turn old, just like what we say in Mandarin, the filial piety. […] I should look after him (the stroke survivor), it is what I need to do (as a son)’. (Louis, non-indigenous family caregiver, son)*

Stroke survivors encountered obstacles in resuming their household roles, while family members adjusted their work to accommodate caregiving responsibilities, resulting in biographical disruption (Bury, 1982). The responsibility of familial support draws two individuals together as a dyad to face the stroke crisis and biographical life transitions as one.

### Acceptance passage (status passage 1)

The acceptance passage manifested in the dyads’ realisation that full recovery was unrealistic. They came to expect to live a changed life with their changed body, which motivated them to participate actively in rehabilitation. Andrew valued rehabilitation more than other healthcare services, stating:

*‘These two parts of care services (vital signs check-up) can be added up to the timeslot of exercise, because exercise is the most important. My expectation is to recover sooner’. (Andrew, indigenous stroke survivor)*

Rehabilitation became an anchor in their everyday lives, as it provided opportunities for physical improvement and psychosocial preparation for discharge arrangement. Clara recounted:

*‘People there kept discussing plenty of information. I knew the LTC services when we stayed in hospitals. At that time, I thought that I must apply for the services with the Rehabus shuttling back and forth to go to rehabilitation. […] I don’t really have any requirements for him, just seeking not to degenerate’. (Clara, non-indigenous family caregiver, wife)*

The rehabilitation programme significantly guided the direction of the acceptance passage by affording time and space for recovery. The dyads accepted the changed body, which was reflected in their behaviour of attaching importance to exercise and seeking to maintain function.

### Sense of interdependence (mindset 2)

The dyads had a sense of interdependence in that they could not go about their lives without each other. Prima described the embodiments of bonded life as follows:

*‘I couldn’t leave, I couldn’t deal with the household chores, couldn’t be away and go on errands. For example, I couldn’t go out to pay the bills because she would phone me once I was away and ask, where are you?’ (Prima, indigenous family caregiver, elder sister)*

The stroke survivors experienced a loss of control over their bodies and social lives, while their family caregivers felt a loss of autonomy over their own flexible time. Both members of the dyad co-experienced a common fate in adapting to the challenges of altered roles and a changed life.

### Alteration passage (status passage 2)

Although familial responsibility linked the dyads into an interwoven life, they then faced an imperative alteration passage, as the family caregivers needed to respond to other responsibilities in the family. Beryl recounted:

*‘I told her, “I want to start working because I feel my husband works hard for earning money by himself. Kids are growing up and expenditure is growing too.”’ (Beryl, indigenous family caregiver, daughter-in-law)*

The alteration passage was driven by expectations that stroke survivors could become more independent. Clara indicated that the LTC services had started to compensate for the care needs of the stroke survivor:

*‘Now he gets a residential care attendant to accompany him when he goes to the rehabilitation appointment. I can have my time; otherwise, I would worry when I am delivering the newspaper whether he fell or not during these couple of hours’. (Clara, non-indigenous family caregiver, wife)*

The dyads engaged with the LTC system to cope with multiple tasks and enable family caregivers to achieve multiple tasks in their lives, both internally in the family and externally within society.

### Sense of independence (mindset 3)

A sense of independence was perceived when the survivors learnt how to exert partial control over their changed bodies to overcome challenges in their lives. Julian was pleased about experiencing control and self-autonomy:

*‘If I was unable to reach some body part (when I took a shower by myself), I just bought a long-handled brush with which I could reach it. […] I felt more… autonomous… that I could control things. I didn’t overly depend on others’ assistance. Otherwise, I would keep waiting’. (Julian, non-indigenous stroke survivor)*

The dyads developed a new relationship as a partially independent life and time management became possible.

### Identification passage (status passage 3)

The sense of split body-self can be regained by applying self-efficacy and establishing self-identity. Andrew used to be a part-time harvesting worker and was now self-sufficient, running a grocery shop with the assistance of LTC services. The family caregiver, Georgia, described how they negotiated the LTC conduct:

*‘It was my job to replenish the stock when my legs were able. But now, I really can’t do it anymore. I’ve been thankful to [have] assistance in showering, tidying up the room, as well as replenishing the stock’. (Georgia, indigenous family caregiver, wife)*

The flexible management of LTC services supported the replenishment work for Andrew’s grocery business and alleviated the load of his family caregiver. Moreover, the participants’ physical and social relationships within the environment could also anchor or hinder their opportunities to re-establish self-identity. Linda recalled how indigenous and cultural identity had influenced her definition of a place, which led to challenges with her recovery process in the urban setting:

*‘I psychologically perceived it was better to go back to the mountain. I was able to hear clan members speak the indigenous language. I felt I was doing well in the surroundings because I could hear my own language’. (Linda, urban-based indigenous stroke survivor)*

People establish self-identity through defining places. Indigenous people’s sense of place was attached to their native land, influencing their self-identity reestablishment along the recovery trajectory.

### Wellbeing state (mindset 4)

The dyads managed to come to terms with post-stroke changes and were able to look actively at their life transitions. Julian described his belief about recovery:

*‘The body, mind, and spirit have to align and proceed together, which leads to a better recovery. If only the body makes the effort, while the mind and the spirit keep getting injured, the recovery seems to lack momentum to keep moving forward’. (Julian, non-indigenous stroke survivor)*

The rehabilitation centre provided not only facilities for physical improvement but also a sense of belonging and wellbeing, as Leonard stated:

*‘As classmates, we chat and share our experiences while everyone is doing their rehabilitation activities. The happiest time of my day is the hours I spend there’.(Leonard, non-indigenous stroke survivor)*

The wellbeing of stroke survivors was enhanced through a combination of their beliefs and active participation in social environments. The dyads underwent processes of acceptance, alteration, and identification in their journey towards wellbeing, underpinning biographical continuation. The rehabilitation programme supports accepting changes in body-self and social life. Engaging with the LTC system facilitates independence and alters interpersonal relationships. Active engagement with social communities strengthens self-identification. For the indigenous groups, regaining self-identity and integrating into tribal surroundings are crucial for their recovery.

## Discussion

Stroke occurrence can lead to a biographical disruption, causing the stroke survivor to lose control over their bodies and social lives, while their family caregivers experience a loss of autonomy in everyday life. This study reveals that biographical continuation lies in the stroke survivors regaining control over their changed bodies so that family caregivers’ workload can be alleviated and both of them can regain autonomy. The current study, adopting the concept of biographical disruption (Bury, 1982), contextualises the changed body-self and loss of identity, which enabled the dyads to become aware of taken-for-granted behaviours, rethink their biographies, and mobilise resources for addressing their altered situation. This study traces the post-stroke recovery trajectory and identifies three status passages – acceptance, alteration, and identification. The status passages are momentum statuses driven by purposive actions and behaviours (Glaser and Strauss, 2011). They show how interpersonal dynamics and healthcare resources originating from the dyads’ needs relate to the efficacy of recovery.

The dyads embodied uncertainty, confusion, and adaption at the beginning of the stroke occurrence and discharge to community settings (Hodson et al., 2019). Their recovery and adaption to the changed body were anchored in the circumstances of knowledge, resources, dyadic relationship, and environment. Our findings show that the prolonged rehabilitation scheme granted time and space for the dyads to accept the irreversible sequelae and realise the commonalities of requiring long-term rehabilitation. Their engagement with rehabilitation helped to lessen their knowledge gap, with guidance from health professionals, and reorient their goals to adapting new normalcy instead of returning to their old life (Hodson et al., 2019). The knowledge, preparation, and attitude encompassing the emphasis on physical improvement were the basis for cohesive body-self establishment (Timothy et al., 2016). This also buffered the anxiety of the dyads (Ostwald et al., 2009) and enhanced family caregivers’ commitment and capacity for providing care (Young et al., 2014).

The dyads considered themselves as experiencing a common fate and maintained the ‘biographical we’ (Aasbø et al., 2016) in the family caregiving relationship, linked by filial piety and familial responsibility (Young et al., 2014; Ng et al., 2016). Consistent with previous studies, family caregivers were usually female and often needed to assume childcare tasks and provide financial support in the household (Alpass et al., 2017; Yan et al., 2019). The focus on post-stroke caregiving impacted their availability for performing other roles, such as mother, wife, and breadwinner. The alteration passage stemmed from the dyad’s intention of independence and succeeded via the positive relationship between them. The LTC system became a key driving factor of the transitioning process, as it is the primary resource compensating for the shrinking capacity of the family caregiving system, alleviating the workload of the family caregivers, and addressing some of their individual needs, which provided a sense of protection in everyday life (Kruithof et al., 2016). A positive dyadic relationship is associated with better physical quality of life (QOL) for stroke survivors and improved social and psychological QOL for caregivers (Pucciarelli et al., 2021). Continuity of care and responsive interventions targeting the dyadic approach directed at both patient and family caregiver may help mitigate the loss of self that leads family caregivers to set aside their own needs (Pindus et al., 2018; Lamontagne et al., 2019).

Body and self are not static, but dynamic and dependent on the situation. Stroke survivors tried to strengthen their self-concept by regaining familiarity with their changed bodies and continually testing their boundaries in everyday life. The LTC system workforce made long-term rehabilitation accessible by supplementing the insufficient human resources in the family caregiving system. Rebuilding self-identity through social participation became the focal core of psychosocial recovery following adaptation to motor learning and functional independence. Routines and repeated social interactions with social actors supported by LTC services have the potential to build a renewed sense of identity through attachment to a meaningful place (Ryden, 1993; Rowles and Bernard, 2012). People identified themselves by defining their environment and generating a sense of place (Jorgensen and Stedman, 2001). The stroke survivors in this study re-established their sense of place through functional, emotional, and social attachment. Indigenous people were more likely to apply ethnic and cultural identities to create place attachment, but this was hindered for dyads living in the urban region, which impacted their biographical continuation. This is consistent with findings from Kelly et al. (2022) indicating that indigenous stroke survivors’ preference for healthcare provided by familiar people such as family or known health staff limits their access to adequate quality healthcare. Indigenous-specific attachment to cultural symbols and native lands indicates more granular challenges in organising care for minority groups under the mainstream healthcare system. Involving indigenous people in healthcare policymaking is pivotal, as it enables an inclusive responsive approach to addressing the unique needs of indigenous populations, including flexibility, language, literacy, and cultural awareness (Quigley et al., 2019).

### Limitations

Data analysis was carried out by the first author with input from the supervisory team, who are from majority ethnic groups. This may limit our understanding of the indigenous culture and lead to an overly shallow view, particularly because presenting the analytical results to participants was not applicable within the limited fieldwork time. The participants were accessed by proxy through the LTC providers, which may have resulted in downplaying negative aspects and emphasising positive ones. Although different relationships within dyads can result in different post-stroke experiences, such differences are not apparent in our study. This could be attributed to our research design, which recruited dyads from residential settings receiving LTC, resulting in participants experiencing similar patterns of post-stroke care. To gain insights into diverse post-stroke patterns of life, future research could recruit families who utilise support from live-in foreign care workers or other healthcare resources.

### Conclusion

Biographical continuation is an essential aspect of the recovery of stroke survivors and their family caregivers; it is grounded in accepting the changed body-self, altering interpersonal relationships, and re-establishing self-identity in the community. LTC services play a vital role in facilitating biographical continuation by supporting stroke survivors in social participation and reducing the multitasking burden on family caregivers. For indigenous participants, reintegration into their native community and regaining identity were crucial in determining their quality of recovery. Therefore, integrating post-stroke care into various care contexts and incorporating indigenous-specific needs into policymaking could support dyads in adapting to their communities.

## Supplementary material

For supplementary material accompanying this paper visit http://doi.org/10.1017/S1463423623000610. click here to view supplementary material
