# Supplementary material for: Biographical continuation: recovery of stroke survivors and their family caregivers in Taiwan
Source: Prim Health Care Res Dev. 2024 Jan 5;25:e2. doi: 10.1017/S1463423623000610 (PMC10790715; doi:10.1017/S1463423623000610)
Supplement: Supplementary file 1 [file phcsup.zip › S1463423623000610sup002.docx]

**Supplementary file 2.** **Interview topic guide**

| ***To understand how services may have changed over time:***   - Could you please describe the long-term care (LTC) services you have received? - Which services you currently use? - Could you please describe the daily living after you got home after being discharged from the hospital? - What has changed (in daily living) after the stroke onset?   (If the stroke survivor is incapable of answering this question, I feel like to ask he/she to describe one-day life before and after the stroke respectively.)   - What difficulties you have encountered? - How is the adaptation? - Does any incident happen after returned home?   ***To understand types of assistance or sources he/she might need in daily living:***   - Could you please talk about the assistance you required in the daily living?   ***To understand post-stroke family caregiving at home:***   - How has your family discussed the post-stroke care? - Could you tell me reasons why you serve as family caregiver? - How did you think about the caregiving work? - What difficulties you have encountered? - What do you do within that circumstance? - How did you talk about your care demands to your family?   ***If the stroke survivor experienced resettlement, such as previously institutionalisation, being cared for by another family member,*** ***post-disaster resettlement, etc.:***   - Could you please talk about reasons why you changed the residence? - Could you please describe how life was going there? - How do you perceive the differences between those places? - Could you please talk how your social life has changed after stroke?   ***If the stroke survivor used to institutionalisation:***   - Could you please explain the circumstance/ or the reasons why you choose the institution as your residence? - What makes you return home? Or what makes you resettle at here?   ***To understand the residential care services:***   - How has your life changed after the involvements of the residential care services? - How has your daily life been assisted? - How is now and the period after you went back from hospital after the stroke different?   ***If Rehabilitation is accessible to the dyads:***   - How do you perceive your rehabilitation? - What has changed after accepted the rehabilitation? - Could you please describe the content of your rehabilitation?   Positive feedback🡪Why do you think it is important to accept rehabilitation?  In what ways, the rehabilitation influences your life?  Negative feedback🡪Why do you think that way?  Could you please describe your perceptions about good rehabilitation?  What a good outcome of the rehabilitation should have looked like?  ***To understand perception of attending the LTC stations in the community:***   - Would you describe your experiences of attending the LTC station? - How has life changed after you attending the LTC station?   ***The perceptions of the general LTC services:***   - Where do you know the information of the LTC services? - Would you share what LTC services mean to you? - How has your daily life been assisted? - Could you please describe your perceptions about good care? - How you express your issues when you encounter it? - How the LTC services could be improved   ***If religion or belief has been mentioned, I would like to probe as follows:***   - Could you please explain how religions/ beliefs influence on people in their daily living? - How have religions/ beliefs impacted on people’s attitudes/ ways of thinking after they got sick? |
| --- |
